# Data Mining and in Silico Analysis of Ethiopian Traditional Medicine: Unveiling the Therapeutic Potential of *Rumex abyssinicus* Jacq.

**DOI:** 10.1007/s12013-024-01478-4

**Published:** 2024-08-17

**Authors:** Lemessa Etana Bultum, Gwangmin Kim, Seon-Woo Lee, Doheon Lee

**Affiliations:** 1 Department of Bio and Brain Engineering, Korea Advanced Institute of Science and Technology (KAIST), Daejeon, South Korea; 2 Bio-Synergy Research Center, Daejeon, South Korea; 3 Institute of Agricultural Life Sciences, Dong-A University, Busan, South Korea

**Keywords:** Natural products, Drug discovery, Machine learning/Artificial intelligence, Molecular modeling

## Abstract

Multicomponent traditional medicine prescriptions are widely used in Ethiopia for disease treatment. However, inconsistencies across practitioners, cultures, and locations have hindered the development of reliable therapeutic medicines. Systematic analysis of traditional medicine data is crucial for identifying consistent and reliable medicinal materials. In this study, we compiled and analyzed a dataset of 505 prescriptions, encompassing 567 medicinal materials used for treating 106 diseases. Using association rule mining, we identified significant associations between diseases and medicinal materials. Notably, wound healing—the most frequently treated condition—was strongly associated with *Rumex abyssinicus Jacq*., showing a high support value. This association led to further in silico and network analysis of *R. abyssinicus* Jacq. compounds, revealing 756 therapeutic targets enriched in various KEGG pathways and biological processes. The Random-Walk with Restart (RWR) algorithm applied to the CODA PPI network identified these targets as linked to diseases such as cancer, inflammation, and metabolic, immune, respiratory, and neurological disorders. Many hub target genes from the PPI network were also directly associated with wound healing, supporting the traditional use of *R. abyssinicus* Jacq. for treating wounds. In conclusion, this study uncovers significant associations between diseases and medicinal materials in Ethiopian traditional medicine, emphasizing the therapeutic potential of *R. abyssinicus* Jacq. These findings provide a foundation for further research, including in vitro and in vivo studies, to explore and validate the efficacy of traditional and natural product-derived medicines.

## Introduction

Over time, numerous cultures worldwide have developed their own traditional healing systems. In recent years, there has been a significant increase in the popularity and acceptance of traditional, complementary, and alternative medicine (TCAM) in both developing and developed countries [1]. Traditional medicine (TM) is widely used and practiced in countries like China, Korea, India, and the African continent [2]. In China, traditional herbal medicine accounts for 30–50% of the total medicinal consumption [3]. In African countries such as Ghana, Mali, Nigeria, and Zambia, herbal medicines are the primary treatment for ~60% of children with high fever caused by malaria [4]. This trend reflects the growing interest in natural product therapy and research on a global scale.

As a result of this increasing interest and utilization, there has been a corresponding rise in the availability of academic articles and electronic databases focusing on traditional medicines. An example of such a resource is the Compound Combination-Oriented Natural Product Database with Unified Terminology (COCONUT), which consolidates 21,456 oriental medicine prescriptions from Korean, Chinese, and Japanese herbal medicine sources [5]. Additionally, the integrated Ethiopian traditional herbal medicine and phytochemicals database (ETM-DB) encompasses details on 573 multi-component prescriptions derived from combinations of 265 medicinal materials found in Ethiopia [6]. The ETM-DB serves as a valuable resource to augment the knowledge and utilization of traditional medicines in Ethiopia, primarily focusing on their application in natural product-based drug discovery. These databases serve to consolidate and disseminate knowledge, supporting research in natural product-based therapies globally.

To harness the power of the vast and diverse data, several key computational techniques have emerged. Coupled with massive computing resources and extensive virtual libraries of drug-like molecules, these developments are reshaping how we approach drug discovery [7]. Computational drug discovery, also known as in silico drug discovery, employs two primary methodologies: Virtual Screening (VS) and Ligand-Based Drug Design (LBDD), each leveraging computational techniques to accelerate the identification and design of therapeutic compounds [8–10]. VS utilizes computational models to analyze extensive compound libraries and predict their ability to bind with target biomolecules associated with diseases. This method helps prioritize compounds based on their predicted binding affinities, guiding further experimental validation [11]. Conversely, LBDD relies on computational models built upon known ligands to design new compounds that mimic their structures or properties, aiming to interact effectively with the target [9]. Both methodologies incorporate techniques such as Quantitative Structure-Activity Relationship (QSAR) modeling to predict biological activities and optimize compounds for efficacy and safety profiles [12]. Additionally, Machine Learning/Artificial Intelligence (ML/AI) methods form another category within computational drug discovery [13, 14]. These methods employ predictive modeling to forecast biological activities or adverse effects of compounds, uncovering patterns and correlations crucial for drug discovery. Data mining is one of the ML methods that could be utilized to effectively handle and analyze large datasets and convert into useful knowledge for various decision making. Data mining is the process of extracting implicit, previously unknown, and potentially useful information from data ([15, 16, 17]).

Association rule mining is a fundamental data mining concept extensively investigated to identify associations between itemsets in vast databases. Its objective is to identify sets of items that frequently occur together in transactional databases. The association rule mining algorithm was first introduced by Agrawal et al., [18], primarily applied to analyze basket data models, where each basket represents a transaction, and the items purchased within the basket are termed as itemsets. Network analysis is another crucial approach that leverages computational technologies to support drug discovery efforts. Biological networks, which represent complex systems of interacting entities, can be categorized into several types, including Protein-Protein Interaction (PPI) networks, signal transduction networks, and gene regulatory networks. To analyze these networks, various methods and algorithms are employed, including centrality measures, community detection, and pathway analysis. The Random - Walk with Restart (RWR) algorithm is particularly valuable for identifying key nodes and functional modules within networks and is often applied to centrality measures and community detection. RWR simulates a stochastic process where a “walker” traverses the nodes of a graph, occasionally returning to the starting point, which aids in exploring the network’s structure and revealing important relationships and functions [19].

Ethiopia is rich in cultural diversity, encompassing over 80 ethnic groups, each with unique traditions and knowledge in traditional healthcare. The country’s history is deeply intertwined with the use of traditional medicine, which encompasses a wide range of practices, including preventive, curative, and even surgical methods, showcasing the diverse and vibrant Ethiopian cultures [20]. According to the New Partnership for Africa’s Development, a significant portion of the Ethiopian population, estimated between 60 and 79%, still relies on indigenous traditional medicine for their healthcare needs [21]. This continuing reliance on traditional medicine can be attributed to various factors. As a developing nation, access to modern healthcare remains limited in many parts of the country. Moreover, the expenses associated with modern medical services make them unaffordable for many people. In contrast, traditional practices are more familiar and culturally accepted, leading to their continued usage. Beyond the cultural significance, Ethiopia’s natural resource abundance contributes to the popularity of traditional medicine practices. The country is blessed with a remarkable diversity of flora, including 6500–7000 floral species, of which 10–12% are exclusive to Ethiopia. Remarkably, more than 1000 plant species, accounting for 15% of the total flora, are utilized for medicinal purposes [22–24]. In this way, Ethiopia’s heritage, cultural acceptance, and abundant natural resources all converge to sustain the utilization of traditional medicine, bridging the gap in healthcare access and meeting the needs of its people.

Traditional medicine in Ethiopia holds significant cultural and health importance, drawing knowledge from diverse sources including ancient records and oral traditions [24]. Indigenous practitioners pass down this wisdom, forming the foundation of Ethiopian traditional literature. Academic research complements this by exploring ethnobotany, ethnomedicine, phytochemistry, and the biological properties of medicinal plants [6, 25]. This heritage plays a pivotal role in Ethiopian society, deeply embedded in its cultural, social, and economic fabric over centuries. Particularly in rural and remote areas with limited access to modern healthcare, traditional medicine serves as a readily accessible and affordable alternative. Its holistic approach, addressing physical, spiritual, and emotional well-being, resonates with local beliefs in comprehensive healthcare. Increasingly recognized for its role alongside modern healthcare, traditional medicine supports chronic disease management and palliative care. Ethiopia’s rich biodiversity provides an abundant source of medicinal plants crucial for traditional remedies, preserving both natural resources and local livelihoods through sustainable practices. Beyond national borders, Ethiopian traditional medicine attracts global interest for its potential contributions to global health solutions. In summary, Ethiopian traditional medicine embodies cultural preservation, community health, and sustainable practices, offering insights and solutions that resonate locally and internationally [26, 27].

Ethiopian traditional medicine, like other traditional systems such as oriental medicine, predominantly involves the use of multicomponent prescriptions. These practices recognize the synergistic interactions and potentiating effects among the various medicinal materials used in prescriptions [28]. However, the available information on the therapeutic use of multicomponent prescriptions remains limited, despite the relatively abundant modern literature on the therapeutic use of individual medicinal materials. It is evident in Ethiopia, as well as in other countries and regions of the world, including India [29], that the presence of diverse cultures and ethnic groups with their own traditional medicine practices leads to variations in prescriptions and constituent medicinal materials for similar symptoms. These prescriptions vary among different traditional medicine practitioners, influenced by cultural practices and geographical locations. Additionally, the availability of specific medicinal materials for prescriptions is influenced by the geo-climatic conditions of a given area, further contributing to these variations.

These variations in traditional medicine practices lead to challenges in establishing a consistent and reliable selection of therapeutic medicinal materials and developing effective prescriptions for treating prevalent diseases. The lack of standardization not only complicates scientific validation but also poses challenges in ensuring the safety and efficacy of treatments, potentially leading to unpredictable outcomes [30, 31]. These challenges have significantly hindered the development of reliable therapeutic medicines, creating barriers to the systematic study and clinical testing needed for modern drug development. To address these issues and promote harmonized practices, standardizing traditional medicine practices through systematic analysis and harmonization is crucial. Our approach in this study aims to identify consistent and reliable medicinal materials, which can then be developed into standardized therapeutic agents. Such an approach not only acknowledges the cultural significance of traditional medicine but also enhances its credibility and applicability in contemporary medical practice.

In this study, we employed data mining and network analysis techniques to analyze prescription data, aiming to establish a reliable collection of medicinal materials for practitioners by identifying associations between common diseases and these medicinal materials. Furthermore, we conducted in silico and network analysis to support these associations and affirm the traditional therapeutic uses of medicinal materials. This included molecular-level investigations to uncover therapeutic targets, pathways, biological processes, and disease associations, exemplified by our analysis of *Rumex abyssinicus* Jacq. (Polygonaceae) and its application in wound healing. By compiling and analyzing traditional medicine data in this manner, our study fills a gap in research by providing comprehensive insights into Ethiopian traditional medicine prescription and supports the development of harmonized and effective natural product-derived medicines.

## Material and Methods

### Compilation and Pre-Processing of Traditional Medicine Prescriptions

Multicomponent traditional medicine prescriptions, derived from natural products, are widely practiced in Ethiopia. Despite substantial literature existing on the therapeutic use of individual medicinal materials, information about the uses of multicomponent prescriptions is limited. For this study, prescription data were manually curated from various sources including research articles, books, and natural product databases. The majority of the prescription data was compiled from the book “Medicinal Plants and Enigmatic Health Practices of Northern Ethiopia” [32], which is the most comprehensive source containing extensive records of traditional multicomponent medicine prescriptions from northern Ethiopia. Authored by expert traditional medicine practitioners, the book draws from comprehensive sources including ancient manuscripts and microfilms from the British Library, which were taken from Ethiopia long ago. Additional prescription information was sourced from research articles focusing on detailed prescriptions from diverse regions of Ethiopia [33–35] and the natural product database, ETM-DB [6].

Despite the scarcity of information on the therapeutic uses of multicomponent prescriptions, the dataset we compiled is sufficient to draw conclusions regarding patterns in medicinal material usage within traditional medicine prescriptions. In this study, prescriptions were selected if they included the use of multicomponent medicinal materials for treating human diseases. To ensure data relevance and specificity, prescriptions for non-illness practices (e.g., remedies for the evil eye, attack deterrents, love potions, and intelligence boosters) were excluded from the dataset. Furthermore, diseases with a limited number of prescriptions and associated medicinal materials were excluded to facilitate effective association rule analysis. The study focused on diseases with a substantial number of prescriptions and diverse types of medicinal materials across prescriptions to identify and analyze consistent medicinal materials for each disease. As a result, the dataset used in this study comprised 505 prescriptions involving combinations of 567 medicinal materials used to treat 106 distinct human diseases.

To conduct association rule mining and identify significant associations between medicinal materials and diseases, we constructed a binary matrix. Each prescription was represented as a row in the matrix, with columns corresponding to human diseases and medicinal materials. The presence of a medicinal material in a prescription was denoted by “1”, while its absence was denoted by “0”.

After identifying significant associations between diseases and medicinal materials, we conducted an in silico analysis, focusing on *R. abyssinicus* Jacq. This analysis involved integrating data on the phytochemical constituents of *R. abyssinicus* Jacq. and therapeutic targets from databases such as the Integrated Ethiopian Traditional Herbal Medicine and Phytochemicals Database (ETM-DB) [6] and the Compound Combination-Oriented Natural Product Database with Unified Terminology (COCONUT) [5]. We explored pathways and biological processes using Kyoto Encyclopedia of Genes and Genomes (KEGG) pathways [36] and The Gene Ontology (GO) database and informatics resource [37].

Additionally, we conducted network analysis by applying the Random- Walk with Restart (RWR) algorithm within the CODA network to identify diseases associated with these targets. Furthermore, we constructed a Protein-Protein Interaction (PPI) network using the CODA network to identify hub targets linked to the phytochemicals. Subsequently, molecular docking and molecular dynamics simulations were carried out to investigate the interactions between the phytochemicals and the identified hub targets, providing deeper insights into their potential therapeutic effects.

**Fig. 1:**
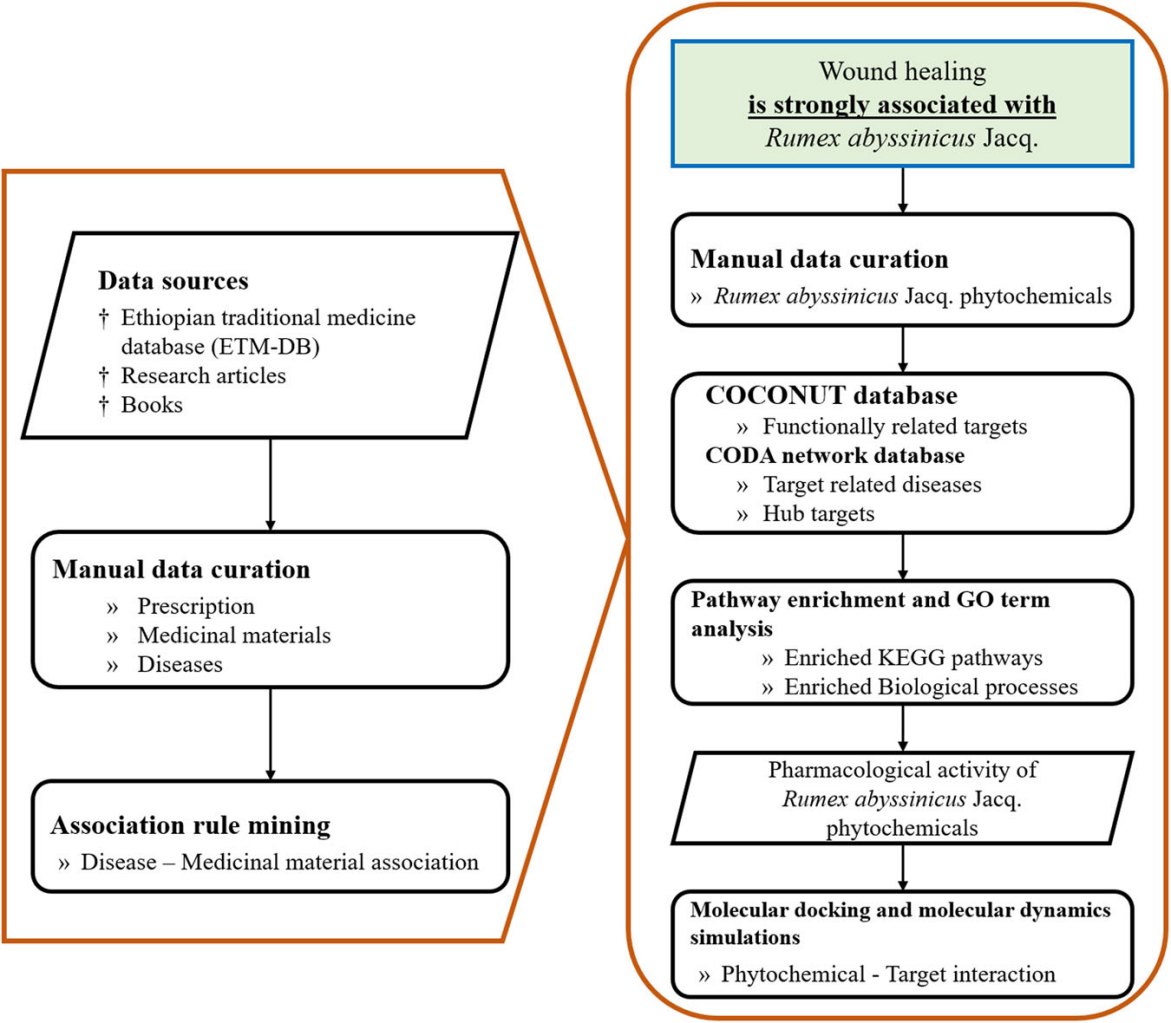
Overview of the process to identify associations between medicinal materials and human diseases from prescription data. Multicomponent prescriptions, constituent medicinal materials, and related diseases were organized in a transaction database. Association rule mining was then employed to extract significant associations between medicinal materials and diseases from the multicomponent prescriptions

Figure 1 outlines the research process from data collection and association rule mining to identify relationships between diseases and medicinal materials. It also depicts the subsequent in silico and network analysis used to explore the therapeutic effects of *R. abyssinicus* Jacq. phytochemicals.

### Association Rule Mining

Association Rule Mining is a key data mining technique used to identify frequent itemsets and their associations in large databases. Originally introduced by Agrawal et al. [18] for analyzing basket data models, it identifies sets of items that frequently occur together in transactions. In this study, we adopted the association rule mining algorithm from Agrawal et al., using user-defined minimum support and confidence thresholds to generate relevant association rules.

For an association rule X (antecedent) ⇒ Y (consequent), the support of the rule is calculated as the proportion of transactions in the data that contain both antecedent and consequent, representing the frequency of occurrence of the rule.

$${\rm{Support}}\left({\rm{X}}\Rightarrow {\rm{Y}}\right)=\frac{{\rm{Number\; of\; transactions\; containing\; both\; X}}\wedge {\rm{Y}}}{{\rm{Total\; number\; of\; transactions}}}$$

Confidence is a measure of how often a rule is true in the data. It is the ratio of the number of transactions that contain both the antecedent and the consequent of the rule, to the number of transactions that contain only the antecedent [38]. A high confidence means that the rule is reliable and consistent. A confidence value of 0 indicates that none of the transactions with the antecedent itemset have the consequent itemset, while a confidence value of 1 signifies that all transactions with the antecedent itemset include the consequent itemset.

$${\rm{Confidence}}\left({\rm{X}}\Rightarrow {\rm{Y}}\right)=\frac{{\rm{Number\; of\; transactions\; containing\; both\; X}}\wedge {\rm{Y}}}{{\rm{Number\; of\; transactions\; containing\; X}}}$$

Lift is a measure of how much more likely the consequent of a rule is to occur when the antecedent is present, compared to when it is absent. It is the ratio of the confidence of the rule, to the frequency of the consequent in the whole dataset [39]. For example, for the association rule X (antecedent) ⇒ Y (consequent), the lift is the confidence of the rule, divided by the proportion of transactions that have Y. A high lift value indicates a significant and interesting rule, highlighting a strong association between the antecedent and the consequent. The lift value of 1 means that antecedent and consequent itemsets are completely independent, which can be achieved by the pure random rules, and the lift value of 2 means that antecedent and consequent itemsets are two times more dependent than the pure random rules [39].

$${\rm{lift}}\left({\rm{X}}\Rightarrow {\rm{Y}}\right)\,=\,\frac{\left({\rm{Number}}\; {\rm{of}}\; {\rm{transactions}}\; {\rm{containing}}\; {\rm{both}}\; {\rm{X}}\wedge {\rm{Y}}\right)/\left({\rm{Number}}\; {\rm{of}}\; {\rm{transactions}}\; {\rm{containing}}\; {\rm{Y}}\right)}{{{\rm{Fraction}}\; {\rm{of}}\; {\rm{transactions}}\; {\rm{containing}}\; {\rm{Y}}}}$$

In this study, association rule mining was employed to identify the associations between diseases and therapeutic medicinal materials using the *arules* package with the *apriori* algorithm in R [40]. The association rules took the form of “antecedent ⇒ consequent”, where the antecedent represented the disease and the consequent represented the medicinal material. Statistical parameters, including support, confidence, and lift, were used to assess the significance of the associations. In this analysis, we set minimum support and confidence thresholds of 0.3 and 10%, respectively. Any generated rule that met or exceeded these thresholds for both support and confidence was deemed statistically significant.

When configuring the association rule parameters for this specific analysis, the support of an item (either disease or medicinal material) is determined by the fraction of transactions (in this context, prescriptions) in which the item is present. The support value of an association rule is calculated as the ratio of the number of prescriptions containing both the disease and medicinal materials to the total number of prescriptions. The confidence value of an association rule is calculated as the ratio of prescriptions that include both the disease and the medicinal material to the prescriptions that include the disease alone. This value provides an indication of how often the rule has been observed to be true. The lift is a measure of how much more likely the medicinal material occurs when the related disease is present, compared to when the disease is absent [41]. It indicates the dependence between the disease and medicinal material. In this analysis, a high lift value and lower confidence suggest that the medicinal material is specifically used for treating that particular disease. On the other hand, a high confidence value and relatively low lift value indicate that the medicinal material is not specific to the disease, and it is utilized to treat various diseases in addition to the associated disease.

**Fig. 2:**
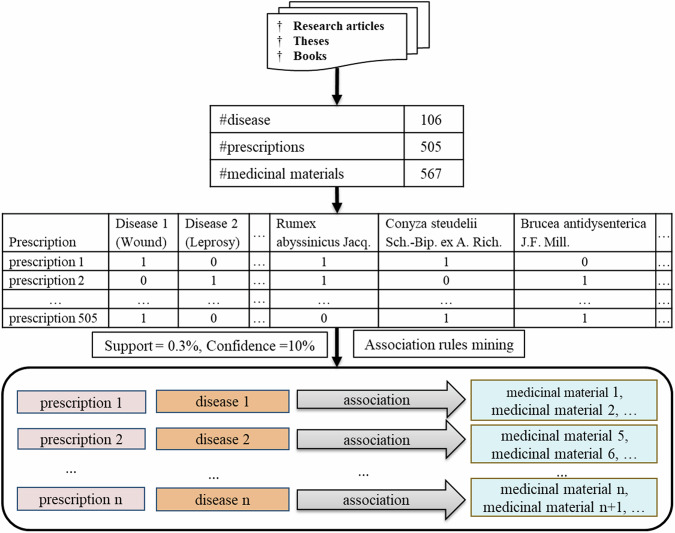
Process for identifying associations between medicinal materials and diseases. It shows the organization of prescription data in a transaction database and the application of association rule mining to reveal significant associations

Figure 2 provides an overview of the process used to identify associations between medicinal materials and human diseases from prescription data. It shows how multicomponent prescriptions, constituent medicinal materials, and related diseases were organized in a transaction database. Association rule mining was then applied to extract significant associations between medicinal materials and diseases from these multicomponent prescriptions.

### Network Visualization of Associations between Traditional Medicine and Diseases

In our analysis, we represented disease and medicinal materials as nodes in a bipartite network, where edges indicate that the medicinal material is part of the prescription used to treat the disease. By employing the Python NetworkX library version 2.5, we constructed, analyzed, and visualized the networks, allowing for a better understanding and visualization of the associations between the interacting items.

To further elucidate these associations, radar charts were employed to visually interpret the association rules between diseases and medicinal materials. These charts effectively present multidimensional data, allowing for a comparison of statistical measures such as confidence and lift across various disease-medicinal material pairs [42]. Radar charts illustrated confidence values for different medicinal materials for selected diseases, depicted lift values indicating the specificity of associations, and presented standardized values for comparative analysis. This visual representation complemented the quantitative analysis by providing insights into the relative significance of medicinal materials in treating specific diseases.

### Exploring *R. abyssinicus* Jacq. Phytochemicals’ Pharmacological Activity Through Target Identification, Network Analysis, KEGG Pathways, and GO Terms

In our study, we explored the pharmacological potential of phytochemicals from *R. abyssinicus* Jacq., with a focus on their therapeutic targets and related pathways [43]. Mukherjee et al., [43]. We specifically targeted *R. abyssinicus* Jacq. because of its notable association with wound healing, as detailed in Table 1.

**Table 1:** Associations between diseases and medicinal materials, including support, confidence, and lift values

Disease	Medicinal Material	Support (%)	Confidence (%)	Lift
Wound	*Rumex abyssinicus* Jacq.	1.39	17.95	5.33
	*Conyza steudelii* Sch.-Bip. ex A. Rich.	1.19	15.38	5.55
	*Plumbago zeylanica* L.	0.99	12.82	2.02
	*Calpurnia aurea* (Ait.) Benth.	0.79	10.26	2.35
Mental illness	*Clerodendrum myricoides* (Hochst.) R. Br. ex Vatke	1.58	29.63	3.48
	*Ruta chalepensis* L.	1.19	22.22	3.87
	*Clausena anisata* (Willd.) Benth.	0.79	14.81	4.68
	*Trigonella foenum-graecum* L.	0.59	11.11	7.01
	*Cannabis sativa* L.	0.59	11.11	5.10
	*Withania somnifera* (L.) Dunal	0.59	11.11	2.81
	*Gladiolus psittacinus* Hook.	0.59	11.11	3.74
	*Verbena officinalis* L.	0.59	11.11	2.95
	*Lepidium sativum* L.	0.59	11.11	2.95
	*Asparagus africanus* Lam.	0.59	11.11	2.44
	*Securidaca longepedunculata* Fresen.	0.59	11.11	2.44
	*Capparis tomentosa* Lam.	0.59	11.11	2.67
	*Adhatoda schimperiana* (Hochst) Nees.	0.59	11.11	2.34
	Myrrh (*Commiphora cf. crenulata* or other *C. spp*.)	0.59	11.11	2.08
	*Cucumis ficifolius* A. Rich.	0.59	11.11	1.22
Sexual stimulation (aphrodisiacs)	*Tragia pungens* (Forssk.) Muell. Arg.	1.19	33.33	28.06
	*Asparagus africanus* Lam.	0.99	27.78	23.38
	*Ferula communis* L.	0.99	27.78	9.35
	*Thalictrum rhynchocarpum* Dill. & A. Rich.	0.79	22.22	4.32
	*Phoenix reclinata* Jacq.	0.59	16.67	7.65
	*Sida cuneifolia* Roxb.	0.59	16.67	7.01
	*Olea europaea* L. subsp. *africana* (Mill.) P.S. Green	0.59	16.67	6.47
	*Gladiolus psittacinus* Hook.	0.59	16.67	5.61
	*Cucumis ficifolius* A. Rich.	0.59	16.67	1.83
	*Habenaria* sp.	0.40	11.11	28.06
	*Sida cuneifolia* Roxb. or *S. ovata* Forssk.	0.40	11.11	9.35
	*Tragia pungens* (Forssk) Muell. Arg.	0.40	11.11	8.02
	*Catha edulis* (Vahl) Forssk. ex Endl.	0.40	11.11	9.35
	*Stylochiton kerensis* N.E. Brown	0.40	11.11	6.23
	*Cannabis sativa* L.	0.40	11.11	5.10
	*Zehneria scabra* (L.f.) Sonder	0.40	11.11	2.67
	*Clerodendrum myricoides* (Hochst.) R. Br. ex Vatke	0.40	11.11	1.30
Eye disease	*Hagenia abyssinica* (Bruce) J.F. Gmel.	0.59	18.75	7.28
	*Premna schimperi* Engl.	0.40	12.50	9.02
	*Artemisia afra* Jacq. ex Willd.	0.40	12.50	7.89
	*Vitis vinifera* L.	0.40	12.50	5.74
	*Solanum marginatum* L.f.	0.40	12.50	5.26
Rabies	*Cucumis ficifolius* A. Rich.	1.39	43.75	4.80
	*Phytolacca dodecandra* L’Herit.	0.99	31.25	10.52
	*Cyphostemma junceum* (Webb) Descoings ex Wild & Drummond	0.79	25.00	10.52
	*Solanum cf. adoense* Hochst.	0.79	25.00	4.86
	*Adhatoda schimperiana* (Hochst) Nees.	0.59	18.75	3.95
	*Sida cuneifolia* Roxb. or *S. ovata* Forssk.	0.40	12.50	10.52
	*Senecio myriocephalus* Sch. Bip. ex A.Rich.	0.40	12.50	7.01
	*Vitis vinifera* L.	0.40	12.50	5.74
	*Kalanchoe petitiana* A. Rich.	0.40	12.50	5.74
	*Mandragora officinarum*/*Luffa aegyptica*	0.40	12.50	4.86
Frequent miscarriage	*Osyris quadripartita* Decne.	0.59	18.75	5.57
	*Clutia abyssinica* Jaub. & Spach.	0.59	18.75	6.31
	*Solanum cf. adoense* Hochst.	0.59	18.75	3.64
	*Cucumis ficifolius* A. Rich.	0.59	18.75	2.06
	*Periploca linearifolia* A. Rich. & Quart.-Dill.	0.40	12.50	10.52
	*Aloe* sp.	0.40	12.50	6.31
	*Rumex nervosus* Vahl	0.40	12.50	4.86
Leprosy	*Plumbago zeylanica* L.	0.99	33.33	5.26
	*Withania somnifera* (L.) Dunal	0.59	20.00	5.05
	*Euclea schimperi* (DC.) Dandy	0.59	20.00	5.61
	*Capparis tomentosa* Lam.	0.59	20.00	4.81
	*Sylvicapra grimmia*	0.40	13.33	16.83
	*Maesa lanceolata* Forssk.	0.40	13.33	22.44
	*Ranunculus multifidus* Forssk.	0.40	13.33	22.44
	*Olea europaea* L. subsp. *africana* (Mill.) P.S. Green	0.40	13.33	5.18
	*Osyris quadripartita* Decne.	0.40	13.33	3.96
	*Clematis simensis* Fresen.	0.40	13.33	5.18
	*Brucea antidysenterica* J.F. Mill.	0.40	13.33	3.96
	*Rumex abyssinicus (Jacq.)*.	0.40	13.33	3.54
	*Jasminum floribundum* R. Br. ex Fresen.	0.40	13.33	3.06
	*Acokanthera schimperi* (DC.) Oliv.	0.40	13.33	3.21
	*Cucumis ficifolius* A. Rich.	0.40	13.33	1.46
Eczema	*Hagenia abyssinica* (Bruce) J.F. Gmel.	0.59	21.43	8.32
	*Cucumis ficifolius* A. Rich.	0.59	21.43	2.35
	*Lichen*	0.40	14.29	14.43
	*Gossypium barbadense* L.	0.40	14.29	12.02
	*Mandragora officinarum*/*Luffa aegyptica*	0.40	14.29	5.55
	*Zehneria scabra* (L.f.) Sonder	0.40	14.29	3.44
Menorrhagia	*Brassica nigra* L.	0.59	21.43	12.02
	*Protea gaguedi* Gmel.	0.40	14.29	36.07
	*Cordia africana* Lam.	0.40	14.29	10.31
	*Nigella sativa* L.	0.40	14.29	6.56
	*Achyranthes aspera* L.	0.40	14.29	3.61
	*Asparagus africanus* Lam.	0.40	14.29	3.14
Malaria	*Dodonaea viscosa* (L.) Jack.	0.59	25	15.78
	*Ekebergia capensis* Sparm.	0.4	16.67	12.02
	*Kanahia laniflora* (Forssk.) R. Br.	0.4	16.67	7.65
	*Jasminum floribundum* R. Br. ex Fresen.	0.4	16.67	3.83
	*Solanum cf. adoens* Hochst.	0.4	16.67	3.24
	*Clerodendrum myricoides* (Hochst.) R. Br. ex Vatke	0.4	16.67	1.96

**Fig. 3:**
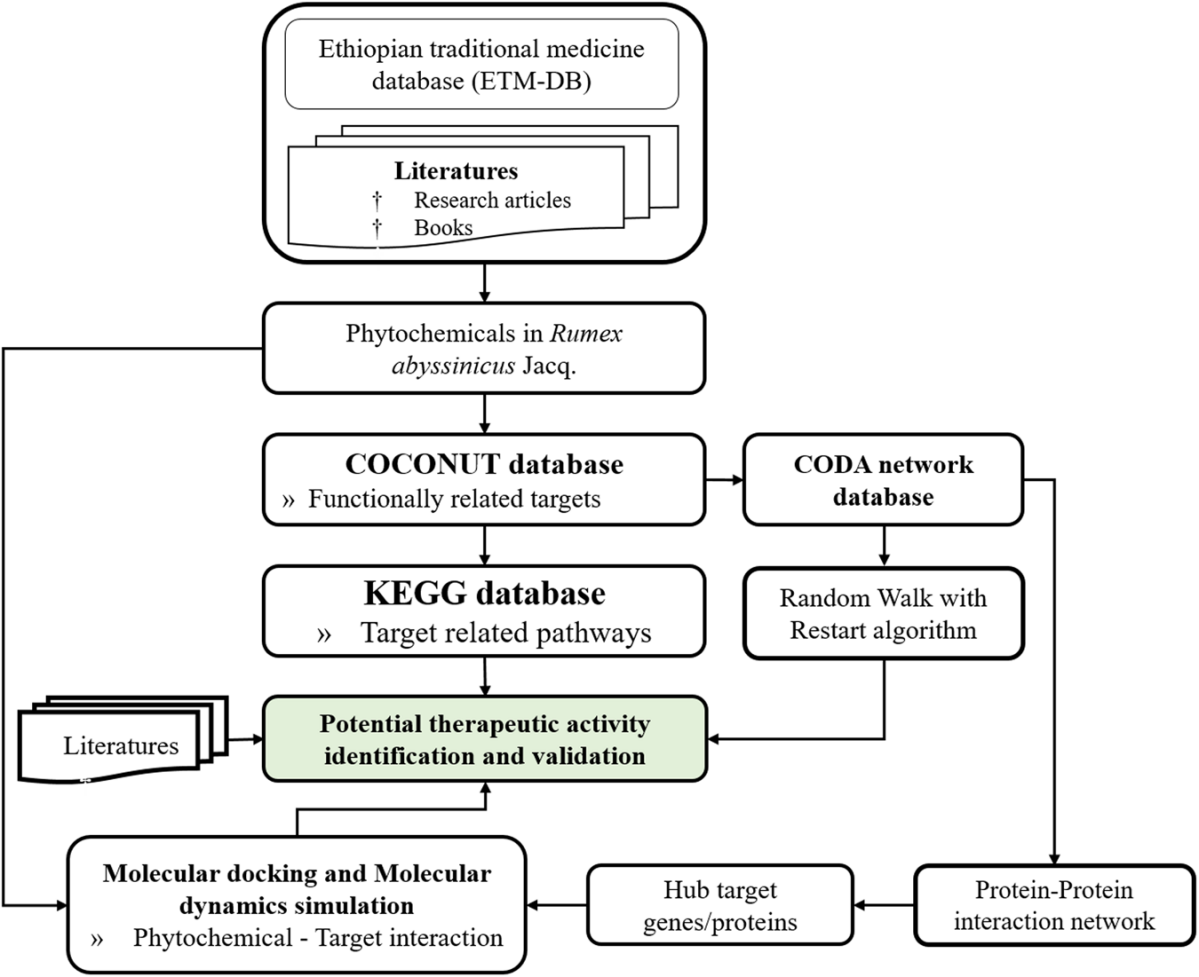
Schematic overview of the methods for identifying potential therapeutic activities of *R. abyssinicus* Jacq. phytochemicals. This process involves compiling phytochemical data from various sources, identifying therapeutic targets using the COCONUT database, constructing a heterogeneous network, and identifying related diseases and hub targets with the CODA network. Pathway enrichment analysis was performed using KEGG and GO terms, followed by molecular docking and molecular dynamics simulations to characterize phytochemical-target interactions

The process outlined in Fig. 3 involved several key steps. First, we compiled phytochemical constituents from sources such as the ETM-DB [6] and relevant literature. Next, we identified functionally related therapeutic targets using the COCONUT database [5], which includes information on traditional herbal medicines, combination drugs, functional foods, and molecular drug/target interactions.

We then constructed a heterogeneous network using the CODA network [44]. Using the RWR algorithm [19], we traced the information flow from therapeutic targets to disease nodes. The RWR algorithm helped us prioritize disease nodes based on their connectivity to the therapeutic targets. Finally, the disease nodes were ranked according to their RWR scores, providing insights into which diseases are most closely associated with the identified targets.

To further understand the interactions of *R. abyssinicus* Jacq. phytochemicals with biological systems, we analyzed the Kyoto Encyclopedia of Genes and Genomes (KEGG) pathway enrichment [36] and Gene Ontology (GO) terms [37] associated with the identified targets. We conducted Gene Set Enrichment Analysis (GSEA) [45] using the enrichr function in the Gseapy Python library (https://www.gsea-msigdb.org/gsea/index.jsp), with gene sets “KEGG_2021_Human”, and “GO_Biological_Process_2021” applying a cut-off of 0.5. This analysis assessed the association of phytochemicals with predefined biological pathways, providing valuable insights into their potential pharmacological effects.

### Integration of PPI Network Analysis, Protein-Ligand Interactions, and MD Simulation in Investigating *R. abyssinicus* Jacq. Phytochemicals

We constructed a Protein-Protein Interaction (PPI) network using the Context-Oriented Directed Associations (CODA) network, which integrates data from multiple sources including BioGRID, KEGG, and EndoNet. The BioGRID data (release 4.4.198) was obtained from https://downloads.thebiogrid.org/BioGRID. For KEGG, we used the REST API available at http://rest.kegg.jp/ to retrieve XML format text files, which we processed to filter protein-protein interactions and convert them into Entrez IDs. Additionally, we accessed data from EndoNet at http://endonet.bioinf.med.uni-goettingen.de in XML format. These data sources were combined, and overlapping interactions were removed. The cleaned data was then used to construct the PPI network with the Python library NetworkX 2.5. The final PPI network comprises 17,358 protein nodes and 261,334 edges.

To focus on therapeutic targets related to *R. abyssinicus* Jacq. phytochemicals, we selected proteins with gene product relationships from this PPI network. We converted the nodes into gene symbols for clarity. From this network, we extracted a hub network highlighting therapeutic targets associated with *R. abyssinicus* Jacq. phytochemicals. Hub targets were identified using two network measures: degree centrality and eigenvector centrality, with hub targets defined as nodes within the top 1% for both measures.

We then performed structure-based molecular docking to evaluate the binding interactions between the phytochemicals and their associated hub targets. For this analysis, we retrieved PDB files for five selected hub proteins from the RCSB Protein Data Bank (https://www.rcsb.org/) and obtained the 3D SDF files of the ligands from the PubChem database [46]. Protein-ligand docking was conducted using the CB-Dock web server (https://cadd.labshare.cn/cb-dock2/index.php), which integrates cavity detection, docking, and homologous template matching. CB-Dock utilizes AutoDock Vina [47]. Finally, we visualized the 3D protein-ligand complexes using CB-Dock, which provides interactive 3D visualizations of the binding modes. We also exported images of these complexes directly from the CB-Dock web server.

Following the molecular docking studies, we conducted Molecular Dynamics (MD) simulations to assess the stability of the binding interactions between the phytochemicals and their target proteins. Emodin, which exhibited a relatively low binding energy score of −8.9 kcal/mol with the target protein ESR1 in docking studies, was selected for the MD simulation. We performed a 100 nanosecond (ns) simulation using the GROningen MAchine for Chemical Simulations (GROMACS) 2021.4 software package [48]. For protein topology preparation, we used SwissParam (https://www.swissparam.ch/) [49], and for ligand topology preparation, we employed the CHARMM27 all-atom force field [50]. The simulation utilized a triclinic box with the Simple Point Charge (SPC) water model and explicit solvent periodic boundary conditions. Charge neutralization of the solvated complexes was achieved with sodium and chloride ions. Energy minimization was performed using the steepest descent method over 5000 steps to eliminate any steric clashes and bad contacts.

A series of equilibration steps followed to relax the system and bring it to an appropriate starting state. This included using a Berendsen thermostat for equilibration under constant Number of particles, Volume, and Temperature (NVT) for 100 picoseconds (ps) [51]. Re-equilibration was carried out with a Parrinello-Rahman barostat, applying a time step of 2 femtoseconds (fs) for each equilibration round over a further 100 ps at constant Number of particles, Pressure, and Temperature (NPT) [52]. The MD production phase was conducted for 100 ns with a time step of 2 fs, maintaining a constant temperature of 300 K and pressure of 1 atm. Using GROMACS’ “trjconv” function, the complex was re-centered and re-wrapped into the unit cells for trajectory analysis. The Root Mean Square Deviation (RMSD) of the protein, ligand, and protein-ligand complex relative to their initial positions, as well as the Radius of gyration (Rg), were used to analyze the trajectories. Data visualization and charting was done using the Grace: GRaphing, Advanced Computation and Exploration of data [53], available at https://plasma-gate.weizmann.ac.il/Grace/.

## Results and Discussion

### Associations between Disease and Medicinal Materials

In this study, we used the a priori algorithm of association rule mining to identify significant associations between diseases and medicinal materials in Ethiopian traditional medicine prescriptions. We assessed these associations using statistical metrics such as support, confidence, and lift. Table 1 presents the support, confidence, and lift values for the association rules between the top ten most frequently treated diseases and their associated medicinal materials, with a focus on rules with a confidence value of at least 10%. For a more comprehensive overview, Supplementary Table S1 online includes all identified associations between diseases and medicinal materials, along with their support, confidence, and lift values.

**Fig. 4:**
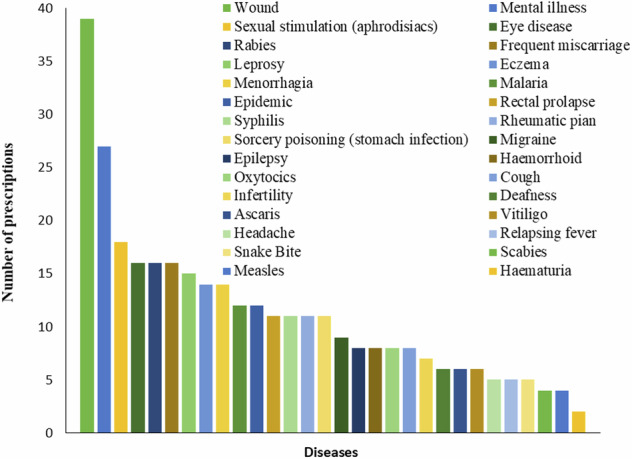
Frequency of various conditions treated with traditional medicine. This bar chart illustrates the prevalence of different conditions addressed by traditional medicine practitioners in the dataset used in this study

To illustrate our findings, we selected particular diseases and their associated medicinal materials based on the frequency of prescriptions. Figure 4 presents a bar chart depicting the frequency of the most commonly treated diseases in our dataset. This chart highlights wound healing as the most prevalent health concern addressed by traditional medicine practitioners. Each bar in the chart represents a specific disease, with its height indicating the number of prescriptions for that disease. Following wound healing, other common conditions include mental illness, sexual stimulation (aphrodisiacs), eye disease, rabies, frequent miscarriage, leprosy, eczema, menorrhagia, malaria, epidemic, rectal prolapse, syphilis, rheumatic pain, sorcery poisoning (stomach infection), migraine, epilepsy, hemorrhoids, oxytocics, cough, infertility, deafness, Ascaris, vitiligo, headache, relapsing fever, snake bite, scabies, measles, and haematuria.

The use of the a priori algorithm allowed us to systematically identify these significant associations, and Table 1 provides a detailed overview of the association rules derived from our analysis.

The significance of the association rules in this study is evaluated using lift and confidence values. Significant associations are indicated by high values of confidence and lift. For instance, the rule wound ⇒ *Rumex abyssinicus* Jacq. shows a high confidence value of 17.95 and a lift value of 5.33. This suggests that *R. abyssinicus* Jacq. is frequently used for treating wounds in traditional medicine. Supporting this, Mulisa et al. [54] demonstrated the wound healing and anti-inflammatory properties of *R. abyssinicus* Jacq. This finding underscores how association rules can identify key medicinal materials, while additional experimental research can validate their traditional uses.

In contrast, the rules Mental illness ⇒ *Clerodendrum myricoides* (Hochst.) R. Br. ex Vatke. and Mental illness ⇒ *Ruta chalepensis* L. exhibit high confidence but relatively low lift values. This indicates that these medicinal materials are frequently used to treat mental illness, but they are also commonly applied to other health conditions. On the other hand, the rule Mental illness ⇒ *Trigonella foenum-graecum* L. has relatively higher lift value of 7.01 and a lower confidence value of 11.11, suggesting that *T. foenum-graecum* L. is more specifically associated with treating mental illness.

For the category of sexual stimulation (aphrodisiacs), the rules Sexual stimulation (aphrodisiacs) *⇒ Tragia pungens* (Forssk.) Muell. Arg., Sexual stimulation (aphrodisiacs) *⇒ Asparagus africanus* (Lam.), and Sexual stimulation (aphrodisiacs) *⇒ Ferula communis* L. show high confidence values, implying these medicinal materials are frequently used as aphrodisiacs. Specifically, *T. pungens* (Forssk.) Muell. Arg. and *Asparagus africanus* Lam. have high confidence and lift values, indicating their prominent role as aphrodisiacs. Conversely, Sexual stimulation (aphrodisiacs) *⇒ Habenaria sp*. has a high lift value of 28.06 and a lower confidence value of 11.11, suggesting that *Habenaria sp*. is more specifically used for aphrodisiac purposes.

Regarding rabies infection, the rule Rabies *⇒ Cucumis ficifolius* A. Rich. shows high confidence (43.75) but relatively low lift values (4.80). This indicates that *Cucumis ficifolius* A. Rich. is commonly used for treating rabies and several other conditions such as frequent miscarriage and sexual dysfunction. In contrast, Rabies *⇒ Sida cuneifolia* Roxb. or *Sida ovata* Forssk. exhibits a high lift value of 10.52 but lower confidence (12.50), suggesting that these medicinal materials are more specifically used for treating rabies infection.

For conditions like frequent miscarriage and menorrhagia, the rules Frequent miscarriage ⇒ *Periploca linearifolia* A. Rich. & Quart.-Dill. and Menorrhagia ⇒ *Protea gaguedi* Gmel. exhibit high lift values (10.52 and 36.07, respectively), indicating their specific therapeutic roles in preventing miscarriage and treating menorrhagia. Further research into these medicinal materials could provide valuable insights into their efficacy.

Patterns observed for leprosy reveal that rules such as Leprosy ⇒ *Plumbago zeylanica* L., Leprosy ⇒ *Withania somnifera* (L.) Dunal, Leprosy ⇒ *Euclea schimperi* (DC.) Dandy, and Leprosy ⇒ *Capparis tomentosa* Lam. have high confidence and low lift values, suggesting these materials are commonly used to treat leprosy and other conditions. Conversely, Leprosy ⇒ *Sylvicapra grimmia*, Leprosy ⇒ *Withania somnifera* (L.) Dunal, and Leprosy ⇒ *Ranunculus multifidus* Forssk. have higher lift values and relatively lower confidence, indicating these medicinal materials are more specifically used to treat leprosy.

Similarly, other significant associations between diseases and medicinal materials can be discerned from Table 1. To substantiate these findings, additional in vitro and in vivo studies are needed to assess the effects of the medicinal materials and their extracts on the associated pathogens and diseases.

### Network Visualization of Associations between Traditional Medicine and Diseases

**Fig. 5:**
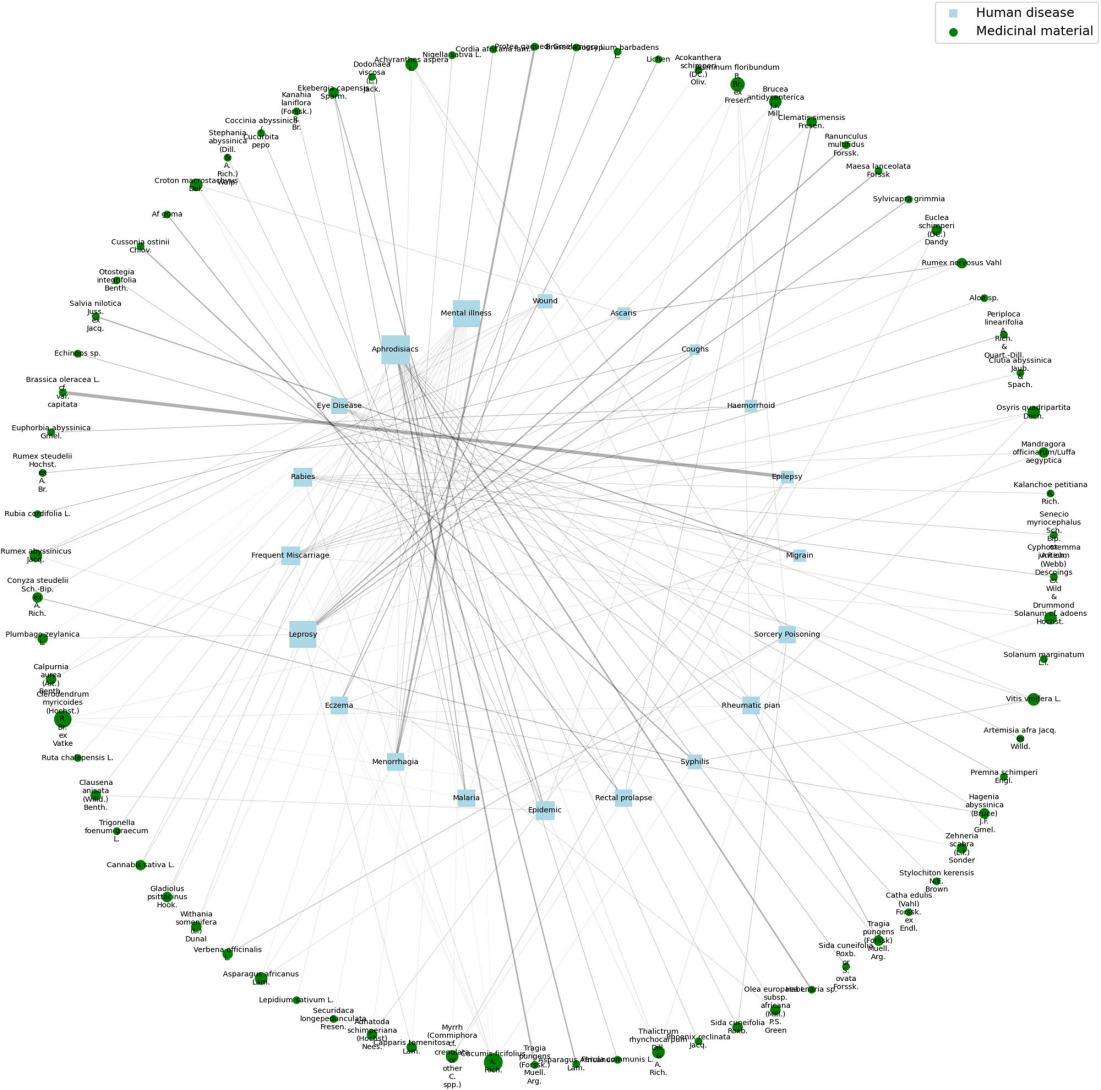
Network diagram illustrating associations between diseases and medicinal materials. The diagram shows 20 diseases (light blue squares) and their associated medicinal materials (green circles). The size of the nodes reflects the degree of each node, and edge width indicates the the specificity of the association (measured by lift)

Figure 5 presents a network diagram that visualizes the associations between 20 different diseases and their corresponding medicinal materials within the dataset used in this study. *Cucumis ficifolius* A. Rich. is shown as a large green circle, indicating its extensive use for treating various diseases. The large light blue square represents aphrodisiacs, highlighting the range of materials used for this purpose. Additionally, the diagram illustrates that leprosy and mental illness are linked to a significant number of medicinal materials, reflecting their diverse treatment approaches.

**Fig. 6:**
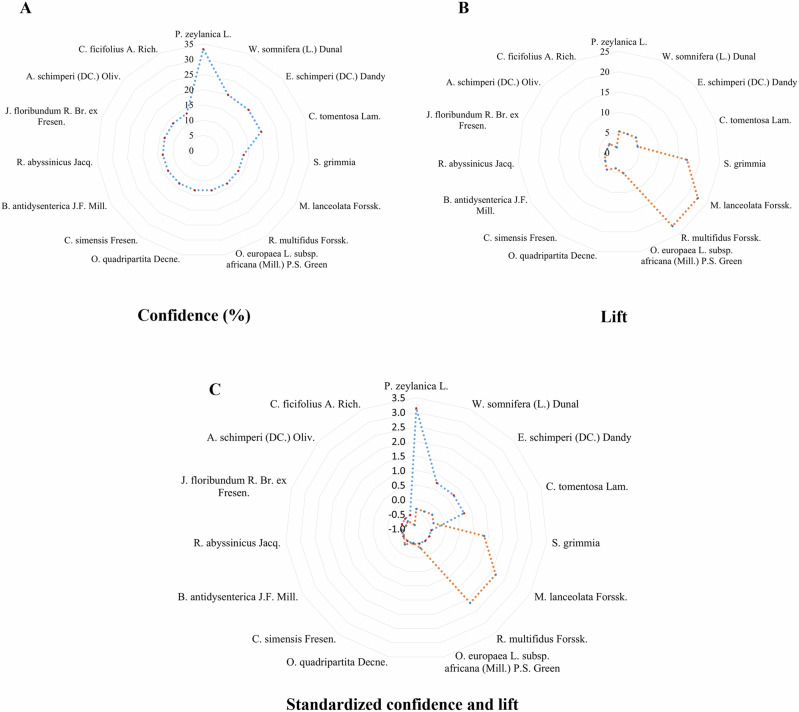
Radar charts illustrating associations between medicinal materials and leprosy. **A** Radar chart of confidence values, indicating the reliability of associations between specific medicinal materials and leprosy. **B** Radar chart of lift values, showing the specificity of these associations, with higher values reflecting materials more specifically used for leprosy treatment. **C** Radar chart of standardized values for both confidence and lift, enabling comparative analysis

Figure 6 shows radar charts to effectively illustrate associations between medicinal materials and associated diseases. Leprosy was selected due to its extensive list of associated medicinal materials, making it a representative example for this visualization method. While these charts focus on leprosy, the same approach can be applied to other diseases. The radar charts show confidence values, which indicate the strength of the associations between specific medicinal materials and leprosy; lift values, which represent the specificity of these associations; and standardized values that facilitate comparative analysis. The charts show that *Sylvicapra grimmia*, *Maesa lanceolata* Forssk., and *Ranunculus multifidus* Forssk. have higher lift values relative to their confidence values, making them particularly specific to leprosy treatment. In contrast, *Plumbago zeylanica* L., *Withania somnifera* (L.) Dunal*, Euclea schimperi* (DC.) Dandy, and *Capparis tomentosa* Lam. display higher confidence values compared to their lift values, indicating these materials are more frequently used to treat leprosy but are also used to treat other conditions.

### Exploring the Pharmacological Activity of *R. abyssinicus* Jacq. Phytochemicals via Target Identification, KEGG Pathways, GO Terms, and Network Analysis

In this study, we manually curated the phytochemicals in *R. abyssinicus* Jacq. From this curated list, we identified ten phytochemicals—emodin, chrysophanol, oleanolic acid, physcion, helminthosporin, lupeol, citreorosein, chrysophanein, and stigmastane-3,6-dione—that are present in the COCONUT database, which provides comprehensive data on compounds and their related human target genes/proteins. Utilizing this extensive compound-target data, we identified 756 human target genes/proteins associated with these phytochemicals.

**Fig. 7:**
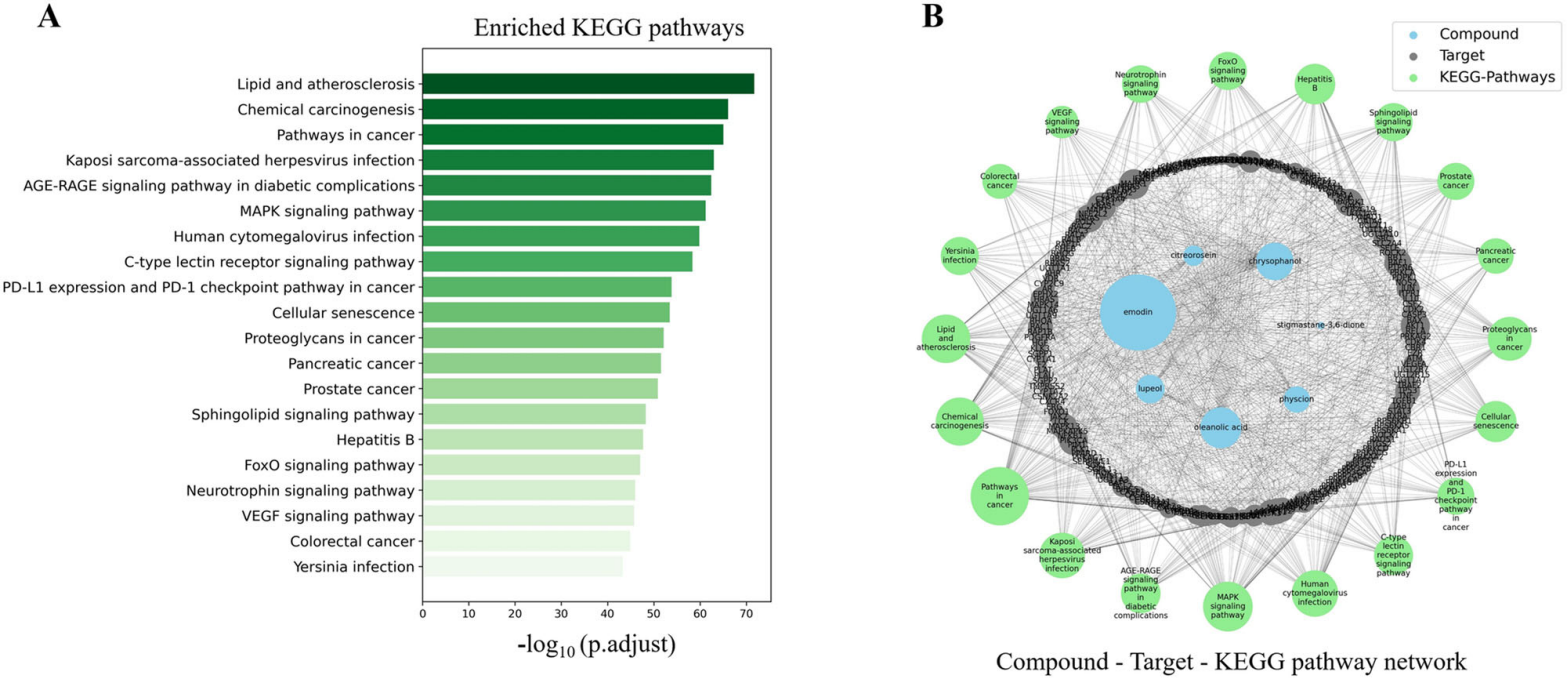
KEGG Pathway Enrichment Analysis. **A** Bar graph showing the top 20 enriched pathways identified through KEGG analysis. **B** Phytochemical-target-pathway network created with Python NetworkX, depicting enriched pathways and their associated genes (Adjusted P ≤ 0.05)

KEGG pathway enrichment analysis of these targets revealed that they are significantly enriched in 196 KEGG pathways (adjusted P-value ≤ 0.05) (Supplementary Table S2 online). Figure 7A highlights the top 20 enriched KEGG pathways, involving 208 target genes/proteins. To visualize these relationships, we constructed a network of targets and pathways using the Python NetworkX package version 2.5, as depicted in Fig. 7B. Key pathways identified include lipid metabolism, atherosclerosis, chemical carcinogenesis, cancer pathways, Kaposi’s sarcoma-associated herpesvirus infection, AGE-RAGE signaling in diabetic complications, MAPK signaling, human cytomegalovirus infection, and C-type lectin receptor signaling. This KEGG enrichment analysis provides valuable insights into the potential therapeutic benefits of *R. abyssinicus* Jacq. phytochemicals. However, further research is needed to elucidate the specific mechanisms through which these phytochemicals exert their effects on the identified molecular targets.

**Fig. 8:**
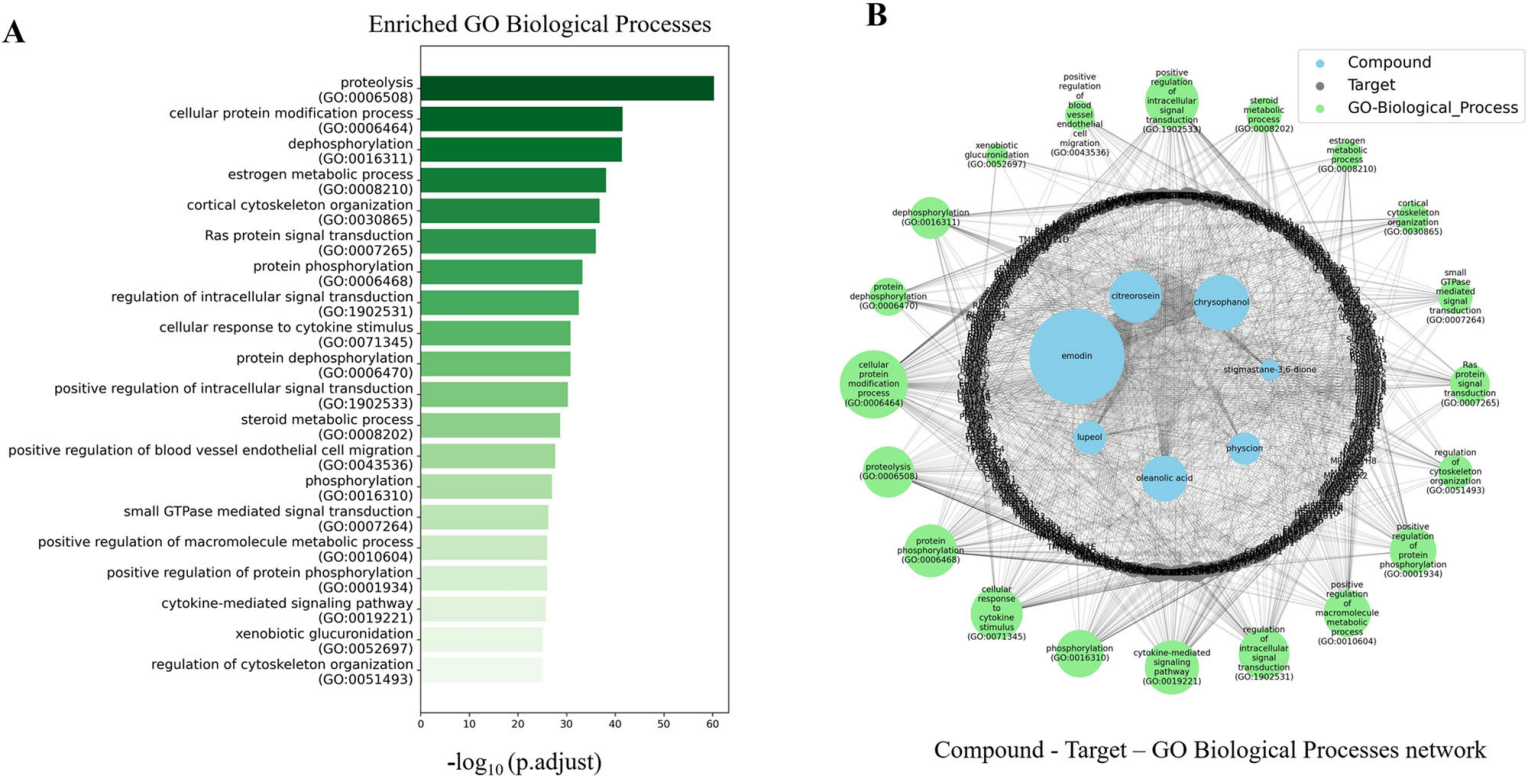
Gene Ontology Enrichment Analysis. **A** Bar graph displaying the top 20 enriched biological processes identified through GO analysis. **B** Phytochemical-target-biological process network generated with Python NetworkX, illustrating enriched biological processes and the genes associated with the phytochemicals (Adjusted P ≤ 0.05)

GO analysis were performed to identify the biological processes associated with the 756 human target genes/proteins related to the phytochemicals from *R. abyssinicus* Jacq. These targets were significantly enriched in 918 biological processes (P-values ≤ 0.05) (Supplementary File S2 online). As depicted in Fig. 8A, the top 20 biological processes primarily involved proteolysis, cellular protein modification, dephosphorylation, estrogen metabolism, cortical cytoskeleton organization, and ras protein signal transduction. Additionally, a phytochemical-target-biological process network was constructed using Python’s NetworkX package (version 2.5). This network, shown in Fig. 8B, highlights that 361 genes are predominantly associated with the top 20 biological processes.

Additionally, a network analysis was performed to identify diseases related to the target genes using the CODA network database employing RWR algorithm (see materials and methods). This analysis linked phytochemicals from *R. abyssinicus* Jacq. to a variety of diseases, including different types of cancers, metabolic and endocrine disorders (such as diabetes mellitus and hypertension), inflammatory conditions, immune system diseases, congenital malformations, respiratory disorders, and nervous system diseases. A comprehensive list of the related diseases is provided in Supplementary Table S3 online.

Many of the diseases identified through the network analysis correspond with the traditional therapeutic uses of *R. abyssinicus* Jacq. For instance, the anti-Alzheimer’s effect of *R. abyssinicus* Jacq. is attributed to its isolated secondary metabolite, helmintosporin, which acts as a dual inhibitor of the enzymes AChE and BChE [55]. Additionally, crude extracts of *R. abyssinicus* demonstrate a broad spectrum of activities, including antibacterial antibacterial [56, 57], anticancer [57], antiviral [56], anti-inflammatory [54, 56], antioxidant [58], wound healing [54], antimalarial [59], diuretic, and analgesic [60] effects.

One objective of this study was to validate the association rule mining results, specifically focusing on the association between wound and *R. abyssinicus* Jacq. To further investigate this association, we conducted in silico and network analysis to explore the molecular-level associations between the phytochemicals in *R. abyssinicus* Jacq. and wound-related conditions. Our analysis indicates that many phytochemicals in *R. abyssinicus* Jacq. are associated with disease categories related to wound, including inflammatory diseases, immune system disorders, and infectious diseases. Inflammation, marked by signs such as heat, redness, pain, swelling, increased body temperature, and fever, constitutes the initial stage of the wound healing process [61]. Infection is also a significant factor that can adversely affect wound healing [62]. These findings provide further support for the traditional use of *R. abyssinicus* Jacq. in treating wounds, aligning with the results obtained from the association rule mining.

### PPI Network, Molecular Docking and Molecular Dynamics Simulations

**Fig. 9:**
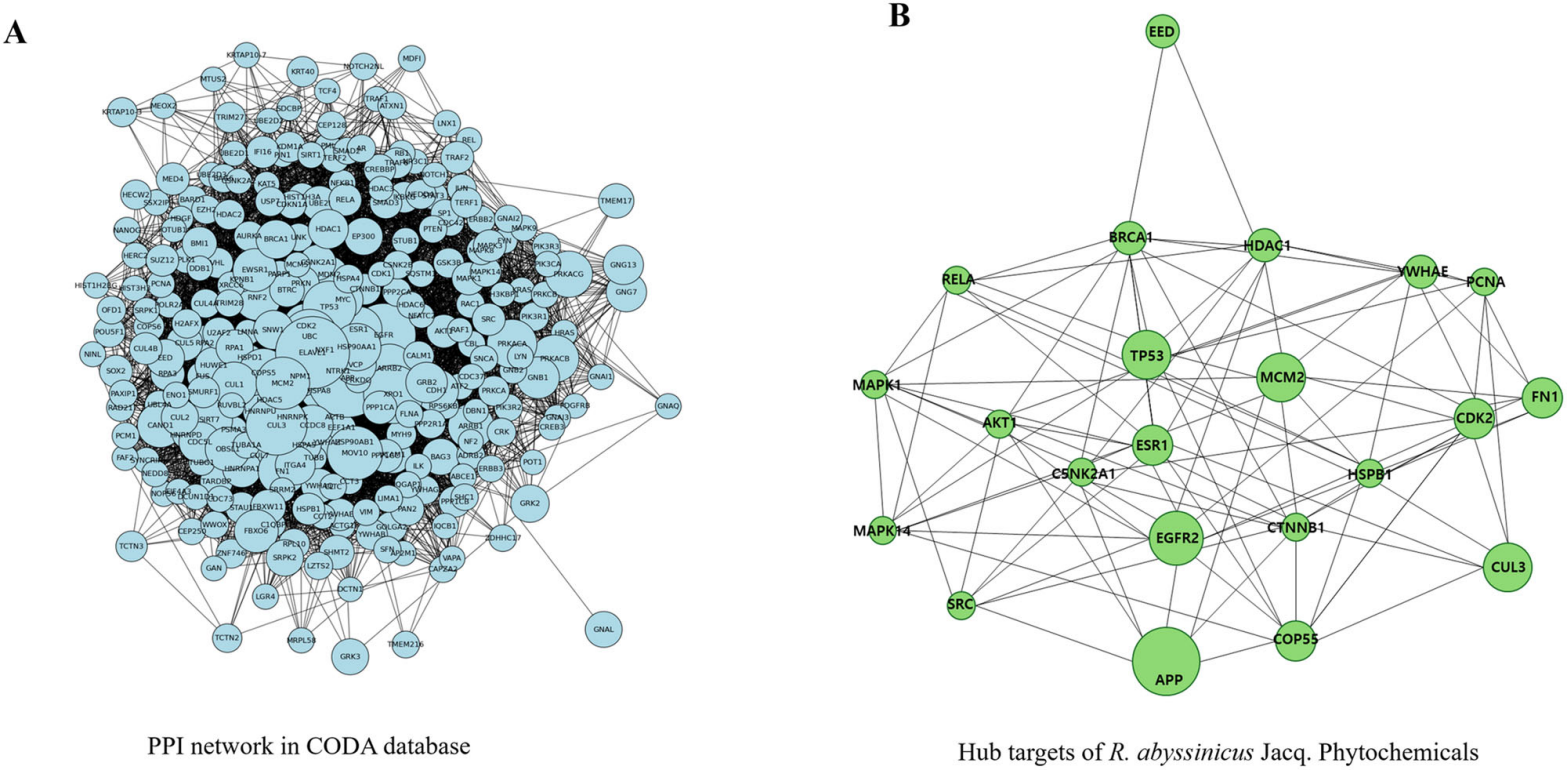
PPI network and hub genes/proteins associated with *R. abyssinicus* Jacq. **A** PPI network constructed using the CODA platform, representing the overall network of protein-protein interactions. **B** Network diagram of the 22 hub genes identified in the top 1% of centrality values from the CODA network, specifically related to the phytochemicals of *R. abyssinicus* Jacq.

**Fig. 10:**
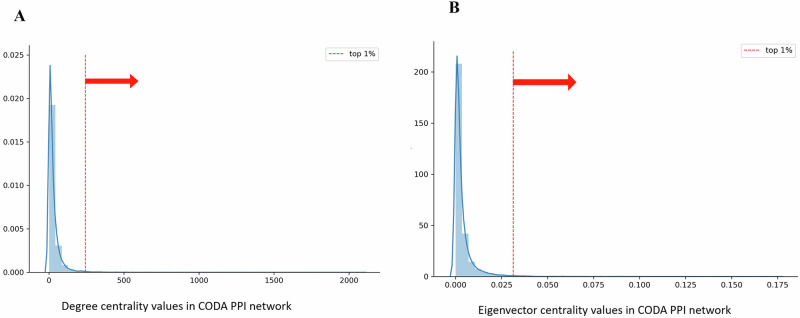
Distribution of centrality values in the CODA network and targets associated with *R. abyssinicus* Jacq. **A** Distribution of degree centrality values across the CODA network, highlighting the top 1% of nodes. Among these, 22 targets associated with *R. abyssinicus* Jacq. were identified. **B** Distribution of eigenvector centrality values across the CODA network, focusing on the top 1% of nodes. The same 22 targets related to *R. abyssinicus* Jacq. were extracted from this subset

From the CODA PPI network (Fig. 9A), we extracted hub targets associated with the phytochemicals in *R. abyssinicus* Jacq., as shown in Fig. 9B. These hub targets, identified through the PPI network, may hold therapeutic potential and offer insights into their clinical value [63]. The distribution of degree and eigenvector centralities for these hub targets was analyzed within the CODA PPI network, which includes 17,358 nodes. The top 1, 5, and 10% of nodes had degree centrality values of 243, 101, and 67, respectively, and eigenvector centrality values of 0.0313, 0.0138, and 0.008, respectively. Notably, 22 targets associated with *R. abyssinicus* Jacq. were found in the top 1% for both degree and eigenvector centralities, as illustrated in Fig. 10A, B.These targets include APP, EGFR, CUL3, MCM2, TP53, COPS5, FN1, ESR1, CDK2, HDAC1, BRCA1, EED, CSNK2A1, AKT1, MAPK1, SRC, YWHAE, RELA, HSPB1, CTNNB1, MAPK14, and PCNA.

Several of these hub genes are linked to the wound healing process. For example, studies have shown that beta-amyloid precursor protein (APP) and its homolog, amyloid precursor-like protein 2 (APLP2), are expressed during skin wound repair in a mouse model of full-thickness skin excision [64]. The EGFR signaling pathway is known to play a crucial role in maintaining skin integrity and promoting wound healing [65]. Additionally, Estrogen Receptor 1 (ESR1) has been found to be enriched in patients with diabetic wounds (DWs) and regulates human skin fibroblasts [65]. The PPI network analysis supports our findings by identifying key hub targets linked to *R. abyssinicus* Jacq. phytochemicals, validating their potential therapeutic value, particularly in wound healing.

**Fig. 11:**
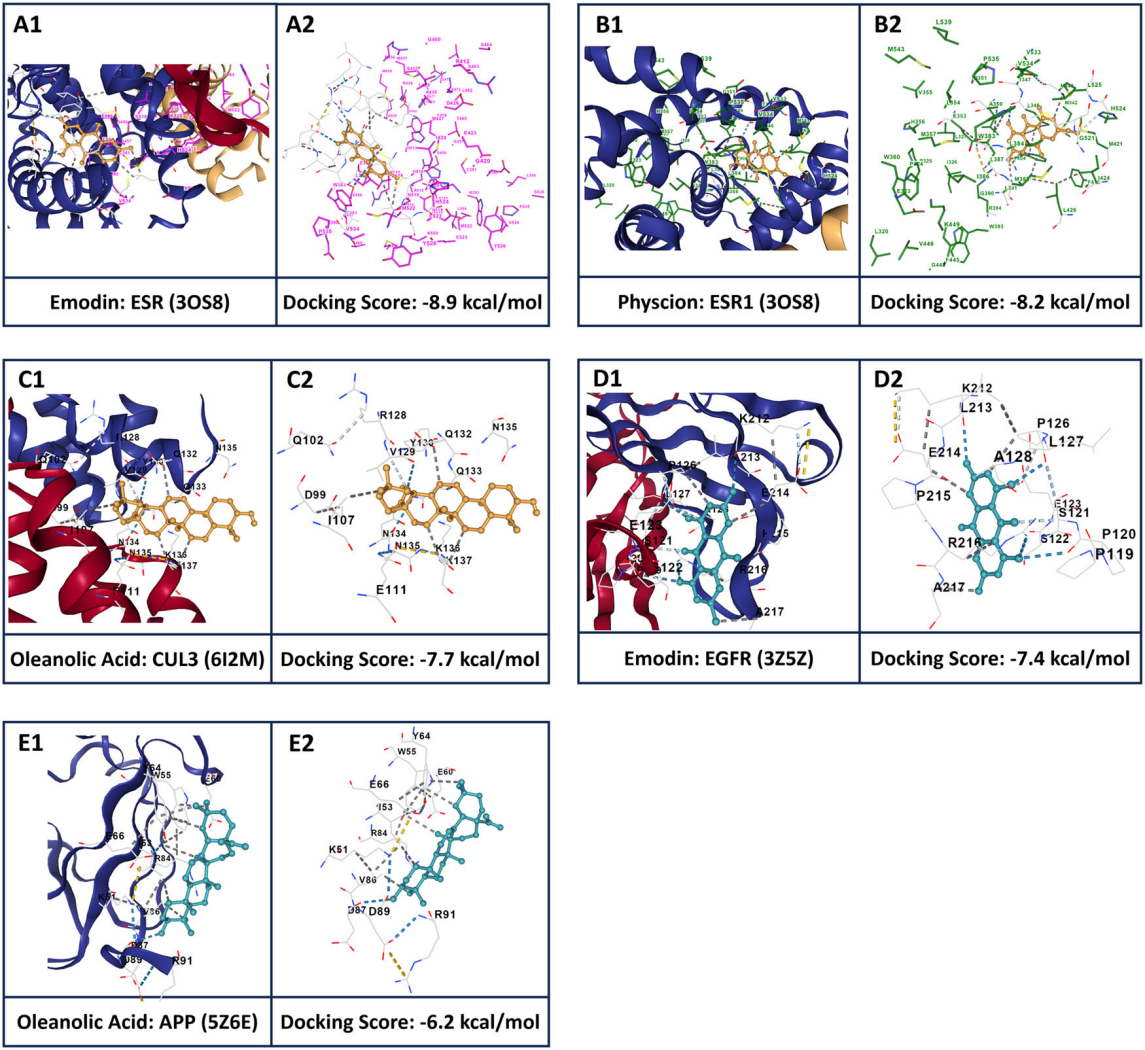
Molecular docking analysis of select phytochemicals from *R. abyssinicus* Jacq. with their corresponding targets. (**A1**–**E1**) 3D visualization of interactions between selected phytochemicals and their corresponding targets. (**A2**–**E2**) 3D pose views of phytochemicals and their interacting residues, with targets removed to clearly show the interaction sites

After identifying hub targets associated with phytochemicals from *R. abyssinicus* Jacq. using the CODA PPI network, we selected a subset of these targets and phytochemicals for further exploration. We performed structure-based molecular docking to investigate their interactions. The docking results indicated successful binding with low binding energies, suggesting interactions between the selected phytochemicals and the hub targets. Figure 11 shows 3D views of these interactions, and Table 2 provides details on binding affinities, interacting amino acid residues, docking centers, and docking sizes for the phytochemicals and their respective receptors.

**Table 2:** Binding affinity energies, interacting amino acid residues, docking center, and docking size for selected phytochemicals from *R. abyssinicus* Jacq. with their receptors

Phytochemical	Target (PDB ID)	Docking Score (Kcal/mol)	Interacting Amino Acid Residues	Docking Center (x, y, z)	Docking Size (x, y, z)
Emodin	ESR1 (3OS8)	−8.9	Chain B: TYR459, THR460, PHE461, LEU462, SER463, ARG515, SER518, ASN519, LYS520, MET522 Chain D: MET343, LEU345, LEU346, THR347, LEU349, ALA350, GLU353, TRP383, LEU384, LEU387, MET388, GLY390, LEU391, ARG394, PRO399, GLY400, LEU403, PHE404, ARG412, GLU423, ILE424, ASP426, MET427, LEU428, LEU429, ALA430, SER433, ARG436, HIS516, LYS520, GLY521, MET522, GLU523, HIS524, LEU525, VAL533	16, 17, −61	35, 32, 31
Physcion	ESR1 (3OS8)	−8.2	Chain D: MET343, LEU346, THR347, LEU349, ALA350, GLU353, TRP383, LEU384, LEU387, MET388, LEU391, ARG394, PHE404, MET421, ILE424, PHE425, LEU428, MET517, GLY521, MET522, HIS524, LEU525, VAL533, VAL534	27, 6, −62	28, 20, 20
Emodin	EGFR (3G5Z)	−7.4	Chain A: PRO119, PRO120, SER121, SER122, GLU123, LEU125, TYR186, PHE209, ARG211, ASN212, GLU213. Chain B: PRO126, LEU127, ALA128, PRO129, GLY130, CYS131, GLY132, ASP133, THR134, THR135, GLY136, VAL139, THR140, LEU141, PRO187, SER188, TRP191, PRO192, LYS212, LEU213, GLU214, PRO215, ARG216, ALA217	11, 39, 50	19, 25, 19
Oleanolic Acid	CUL3 (6I2M)	−7.7	Chain A: GLY41, ALA42, SER43, GLU44, ASP99, GLN102, ILE103, GLY104, SER105, ILE107, THR108, GLU111, ASN134, ASN135, LYS136, LYS137, SER140 Chain B: VAL117, ARG120, MET124, ASP127, ARG128, VAL129, TYR130, VAL131, GLN132, GLN133, ASN135, VAL136, ASN138, VAL139, TYR140	−29, −9, 47	23, 23, 23
Oleanolic Acid	APP (5Z6E)	−6.3	Chain A: VAL27, LYS51, ILE53, TRP55, GLU60, TYR64, GLU66, ARG84, VAL86, ASP87, ASP89, THR90, ARG91	6, 35, 2	23, 23, 23

The binding affinity energies and interactions between *R. abyssinicus* Jacq. phytochemicals and their respective receptors reveal crucial insights into the molecular mechanisms underlying their therapeutic potential. Generally, lower docking scores (more negative values) indicate stronger and more stable interactions. These interactions are primarily driven by hydrogen bonding, hydrophobic interactions, and electrostatic forces. Hydrogen bonds, particularly, enhance the stability and specificity of the ligand-receptor complex, while hydrophobic interactions contribute to the overall binding affinity by reducing the free energy of the ligand-receptor complex [66–68].

Emodin demonstrated the highest binding affinity with ESR1, followed by its interactions with EGFR. Physcione also showed a strong binding affinity with ESR1. These interactions typically involved key residues forming hydrogen bonds and hydrophobic interactions, which stabilized the ligand-receptor complexes and contributed to their high binding affinities. Oleanolic Acid exhibited significant binding affinities with CUL3 and APP. Similar to the other phytochemicals, the interactions were characterized by hydrogen bonds and hydrophobic interactions with key residues, enhancing the stability of the ligand-receptor complexes. Overall, the consistent presence of hydrogen bonds and hydrophobic interactions across these ligand-receptor complexes underlines their significance in determining binding affinity and stability, validating the potential therapeutic applications of these phytochemicals. The docking output and MD simulation results confirmed stable interactions over time, supporting the robustness of these findings [66–68].

We conducted the MD simulation to evaluate the stable binding affinity of phytochemicals with their targets. Emodin, which demonstrated a favorable binding energy score (−8.9 kcal/mol) with the target ESR1 in docking studies, was chosen for the MD simulation. MD simulations provide a dynamic perspective on the interaction between ligands and their targets, complementing the static view offered by docking studies [69].

The RMSD parameter was utilized to examine the stability of the protein-ligand complex over 100 ns. RMSD is a measure of the average distance between atoms of superimposed proteins and is commonly used to assess the stability of protein structures and protein-ligand complexes in MD simulations [70]. The RMSD of the atoms in the ligand, ligand fit to protein backbone, and protein backbone exhibited similar patterns of fluctuation during most of the simulation period. The RMSD of ligand fit to protein backbone and protein backbone displayed an initial increase followed by stability throughout the simulation.

**Fig. 12:**
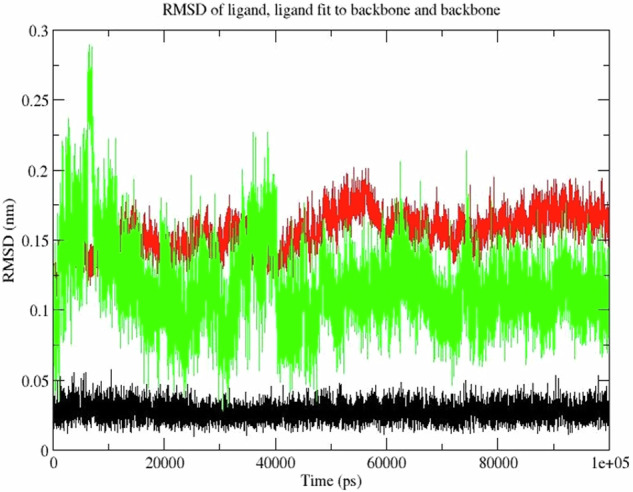
Root Mean Square Deviation (RMSD) plots of ligand and target protein backbone. RMSD plots showing the deviation of the ligand (emodin) (black), the target protein backbone (ESR1) (red), and the ligand fit to the backbone (green) over 100 ns of molecular dynamics simulation

Notably, the RMSD of the ligand (Fig. 12, black) was slightly lower than that of the ligand fit to protein backbone (Fig. 12, green), indicating a conformational change of the target protein in the protein-ligand complex upon binding. This is a common observation in MD simulations, where ligands often induce conformational changes in the protein structure, enhancing the stability of the complex [71, 72]. The protein-ligand complex (Fig. 12, green) remained relatively stable throughout the simulation, indicating tight binding between the ligand and the receptor.

**Fig. 13:**
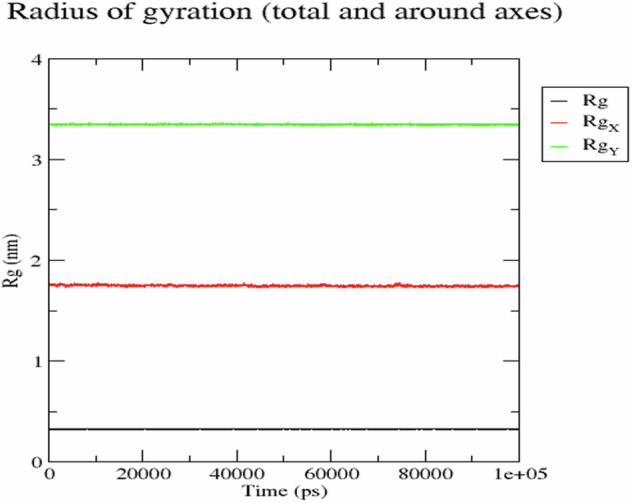
Radius of gyration (Rg) plots. Plots of the radius of gyration (Rg, in nm) for the ligand (emodin) (Rg), the target protein backbone (ESR1) (RgX), and the entire system (RgY) over 100 ns of simulation

Additionally, the Radius of gyration (Rg) analysis confirmed that the protein structures were largely unaffected by the binding region over the simulation period (Fig. 13). Rg measures the compactness of the protein structure and is another indicator of structural stability [71]. The consistent Rg values suggest that the protein maintained its structural integrity upon ligand binding, further supporting the stability of the protein-ligand complex.

Furthermore, the hydrogen bonds formed between the ligand and the protein play a crucial role in the stability of the complex. Hydrogen bonds contribute significantly to the binding energy and specificity of the interactions [73, 74]. The hydrophobic interactions, which reduce the free energy of the system, also play an essential role in maintaining the stability of the complex [75].

These findings suggest a stable interaction between the phytochemical and the target protein, supporting the potential therapeutic significance of the compound. The combined insights from docking studies and MD simulations provide a robust framework for understanding the molecular basis of the observed binding affinities, highlighting the importance of both hydrogen bonding and hydrophobic interactions in stabilizing the protein-ligand complexes [76, 77].

Overall, the integration of docking studies with MD simulations offers a comprehensive approach to validating the stability and potential efficacy of phytochemicals, such as Emodin, in targeting specific proteins. This approach underscores the therapeutic relevance of these compounds in traditional medicine and supports further experimental validation [78].

## Conclusions

Our study aimed to identify and validate significant associations between human diseases and medicinal materials used in Ethiopian traditional medicine prescriptions, ultimately aiming to identify reliable therapeutic medicines for common human diseases. Through association rule mining, we successfully identified notable associations, including a significant association between *R. abyssinicus* Jacq. and wound healing.

To further support these findings, we conducted in silico analysis to explore the molecular-level associations between the phytochemicals in *R. abyssinicus* Jacq. and various therapeutic targets, pathways, and biological processes. This analysis revealed 756 associated targets enriched across diverse KEGG pathways and GO terms, indicating potential therapeutic effects against a broad spectrum of diseases, including cancer, metabolic disorders, endocrine diseases, inflammation, immune system diseases, congenital malformations, respiratory issues, and neurological conditions. Network analysis using the CODA network highlighted key hub target genes linked with *R. abyssinicus* Jacq.‘s phytochemicals, emphasizing their role in wound healing. Molecular docking and molecular dynamics simulations further supported these findings by confirming stable binding interactions between the phytochemicals and the identified hub targets.

In summary, our in silico and network analysis robustly validate the association rule mining results and the traditional therapeutic applications of *R. abyssinicus* Jacq. in wound treatment. However, to fully confirm these results, controlled experimental studies on the pharmacological effects of *R. abyssinicus* Jacq. phytochemicals are necessary [37].

## Supplementary Information

Supplementary Table S1

Supplementary Table S2

Supplementary Table S3

Supplementary Tables Captions

## Abbreviations

CODA: Context-Oriented Directed Associations
COCONUT: Compound Combination-Oriented Natural Product Database with Unified Terminology
ETM-DB: Integrated Ethiopian Traditional Herbal Medicine and Phytochemicals Database
GO: Gene Ontology
GROMACS: Groningen Machine for Chemical Simulations
KEGG: Kyoto Encyclopedia of Genes and Genomes
MD: Molecular Dynamics
ML/AI: Machine Learning/Artificial Intelligence
NPT: Constant Number of Particles, Pressure, and Temperature
NVT: Constant Number of Particles, Volume, and Temperature
PPI: Protein-Protein Interaction
QSAR: Quantitative Structure-Activity Relationship
RWR: Random-Walk with Restart
Rg: Radius of Gyration
RMSD: Root Mean Square Deviation
TCAM: Traditional, Complementary and Alternative Medicine
TM: Traditional Medicine
VS: Virtual Screening
LBDD: Ligand-Based Drug Design
GSEA: Gene Set Enrichment Analysis
