# Supplementary material for: Data Mining and in Silico Analysis of Ethiopian Traditional Medicine: Unveiling the Therapeutic Potential of Rumex abyssinicus Jacq
Source: Cell Biochem Biophys. 2024 Aug 17;83(1):467–88. doi: 10.1007/s12013-024-01478-4 (PMC11870893; doi:10.1007/s12013-024-01478-4)
Supplement: Supplementary file 4 — Supplementary Tables Captions [file 12013_2024_1478_MOESM4_ESM.docx]

**Supplementary Files**

**Table S1.** Associations between diseases and medicinal materials, including support, confidence, and lift values.

**Table S2.** Enriched KEGG pathways and gene ontology (GO) terms for phytochemicals from *R. abyssinicus* Jacq.

**Table S3.** Diseases associated with phytochemicals from *R. abyssinicus* Jacq., identified using the RWR algorithm in the CODA network.
